# Differences in Interleukin-8 Plasma Levels between Diabetic Patients and Healthy Individuals Independently on Their Periodontal Status

**DOI:** 10.3390/ijms19103214

**Published:** 2018-10-18

**Authors:** Petra Borilova Linhartova, Denisa Kavrikova, Marie Tomandlova, Hana Poskerova, Vaclav Rehka, Ladislav Dušek, Lydie Izakovicova Holla

**Affiliations:** 1 Clinic of Stomatology, Institution Shared with St. Anne’s Faculty Hospital, Faculty of Medicine, Masaryk University, Pekarska 664/53, 60200 Brno, Czech Republic; peta.linhartova@gmail.com (P.B.L.); denisakavrikova@gmail.com (D.K.); hana.poskerova@fnusa.cz (H.P.); 2 Department of Pathophysiology, Faculty of Medicine, Masaryk University, Kamenice 5, 62500 Brno, Czech Republic; 3 Department of Biochemistry, Faculty of Medicine, Masaryk University, Kamenice 5, 62500 Brno, Czech Republic; mhyks@med.muni.cz; 4 Faculty of Medicine, Masaryk University, Kamenice 5, 62500 Brno, Czech Republic; vasek.rehka@seznam.cz; 5 Institute of Biostatistics and Analyses, Faculty of Medicine, Masaryk University, 62500 Brno, Czech Republic; dusek@iba.muni.cz

**Keywords:** interleukin-8, chemokines, plasma, polymorphism, chronic periodontitis, diabetes mellitus

## Abstract

Chronic periodontitis (CP) and diabetes mellitus (DM) involve several aspects of immune functions, including neutrophil activity and cytokine biology. Considering the critical function of chemokine interleukin-8 (IL-8) in the inflammatory process, the aims of this study were to determine: (i) IL-8 plasma levels; (ii) *IL-8* (−251A/T, rs4073) and its receptor 2 (*CXCR2*, +1208C/T, rs1126579) polymorphisms, and (iii) the presence of the selected periodontal bacteria in types 1 and 2 DM patients (T1DM and T2DM) and systemically healthy controls (HC) with known periodontal status. This case–control study comprises of 153 unrelated individuals: 36/44 patients suffering from T1DM+CP/T2DM+CP and 32/41 from HC+CP/non-periodontitis HC. Both the clinical and biochemical parameters were monitored. The genotypes were determined using qPCR, IL-8 plasma levels were measured using an ELISA kit. Subgingival bacterial colonization was analyzed with a DNA microarray detection kit. The IL-8 plasma levels differed significantly between non-periodontitis HC and T1DM+CP/T2DM+CP patients (*P* < 0.01). Even in HC+CP, IL-8 concentrations were significantly lower than in T1DM+CP/T2DM+CP patients (*P* ≤ 0.05). No significant associations between the IL-8 plasma levels and the studied *IL-8* and *CXCR2* polymorphisms or the occurrence of selected periodontal bacteria (*P* > 0.05) were found. CP does not influence the circulating IL-8 levels. Patients with T1DM+CP/T2DM+CP had higher circulating IL-8 levels than HC+CP/non-periodontitis HC.

## 1. Introduction

Diabetes mellitus (DM), most frequently occurring as type 1 (T1DM) and type 2 (T2DM), is a chronic metabolic disorder, which impacts global health [1]. The major cause of morbidity and early mortality in diabetic patients stems from vascular complications [2], developing as a consequence of long-term hyperglycemia and the formation of advanced glycation end products (AGE) [3]. The five main complications of DM include retinopathy (DR), neuropathy (DPN), nephropathy (DN), altered wound healing, and macrovascular disease [4]. Chronic periodontitis (CP) is considered the sixth complication of diabetes [5], and it may have an increased influence on systemic levels of cytokines, especially in individuals with T2DM [6].

CP is mainly caused by Gram-negative bacteria in the subgingival biofilm, such as *Porphyromonas gingivalis* and *Tannerella forsythia* [7,8], and by “genetic dysbiosis,” which highlights the role of human genetic variants affecting microbial recognition and host response in creating an environment conductive to changes in the normal microbiota [9]. A common feature in DM and CP patients is a low-grade inflammatory state [10,11], which suggests a link between the two diseases. DM has many adverse effects on the periodontium, including an impaired neutrophil function and the production of cytokines [12].

Interleukin-8 (IL-8), a member of the C-X-C motif (CXC) subfamily of chemokines, is one of the most important chemoattractants and activators of human neutrophils via interaction with two receptors (CXCR1 and CXCR2) [13]. IL-8 is involved in the initiation and amplification of acute inflammatory reaction; it is secreted by several cell types in response to inflammatory stimuli [14]. Chemokines and neutrophils have previously been associated with, or implicated in the pathogenesis of T1DM [15,16,17,18]. Furthermore, the study of non-obese diabetic mouse models has identified CXCR1/2 chemokine receptors as “master regulators” of diabetes pathogenesis [19,20]. Nevertheless, the role of IL-8 in DM and CP pathogenesis remains unclear.

The levels of IL-8 in oral keratinocytes [21], gingival epithelial cells [22], gingival crevicular fluid (GCF) [23,24,25], plasma [21], or serum [26] have been observed with contradictory findings in the context of DM and CP. It is highly probable that *IL-8* (−251A/T, rs4073) polymorphism affects the ability of individuals to produce IL-8 [27]. Furthermore, expression levels of *CXCR2* or specific *CXCR2* gene variants (+1208C/T, rs1126579) have been linked with levels of circulating IL-8 [28,29]. Although no association was previously found between the *IL-8* gene polymorphisms and periodontal diseases in the Czech population, *IL-8* variants have been associated with the presence of some periodontal bacteria as well as specific *IL-8* haplotypes were suggested to be protective against CP development [30].

Considering the critical function of IL-8 in inflammation, and its possible role in the pathogenesis of DM and CP, the aims of this study were to determine (i) IL-8 plasma levels; (ii) *IL-8* (−251A/T, rs4073) and its receptor 2 (*CXCR2*, +1208C/T, rs1126579) polymorphisms; and (iii) the presence of the selected periodontal bacteria in types 1 and 2 DM patients (T1DM and T2DM) and systemically healthy controls (HC) with known periodontal status.

## 2. Results

### 2.1. Study Population

The demographic data for the study population are shown in Table 1. The subgroups did not differ in terms of the male/female ratio (*P* > 0.05). The mean ages and BMI were similar for patients with T1DM+CP and HC, but there were significant differences in mean ages between groups of HC+CP/T2DM+CP and non-periodontitis HC (mean ± standard deviation, SD: 59.5 ± 9.3/66.8 ± 8.5 vs. 45.5 ± 9.6, *P* < 0.01). Mean body mass index (BMI) was significantly higher in T2DM+CP patients than in non-periodontitis HC and T1DM+CP patients (29.9 ± 7.7 vs. 23.8 ± 4.2/25.1 ± 3.1, *P* < 0.05). Smoking status was not different between T2DM+CP and non-periodontitis HC (5.3% vs. 7.1% smokers) or between T1DM+CP patients and HC+CP individuals (19.4% vs. 25.0% smokers). DR was present only in patients with T1DM+CP, and DPN was also present significantly more frequently in this group of patients (*P* < 0.01).

There were statistically significant differences between non-periodontitis HC and all of the subgroups of cases in gingival index (GI) and numbers (*N*) of sites and teeth with a pocket depth (PD) ≥5 mm and an attachment loss (AL) ≥5 mm (*P* < 0.01). Nevertheless, similar numbers were found in mutual comparisons of HC+CP, T1DM+CP, and T2DM+CP patient groups (*P* > 0.05). Although the groups of diabetic patients had similar lipid profiles and blood glucose levels, T1DM+CP patients had poorly controlled glycated hemoglobin (HbA1c) levels when compared to T2DM+CP patients (69.6 ± 12.0 vs. 57.7 ± 14.5, *P* < 0.05). The group of patients with T1DM+CP included only three patients with satisfactory DM control. DM control in other patients was unsatisfactory as measured by HbA1c values. The stratification in the group of patients with T2DM+CP was as follows: 7.7% of patients with well-controlled diabetes, 28.2% with satisfactorily controlled diabetes, and 64.1% with unsatisfactorily controlled diabetes.

### 2.2. IL-8 Plasma Levels and Clinical Parameters

**Figure 1:**
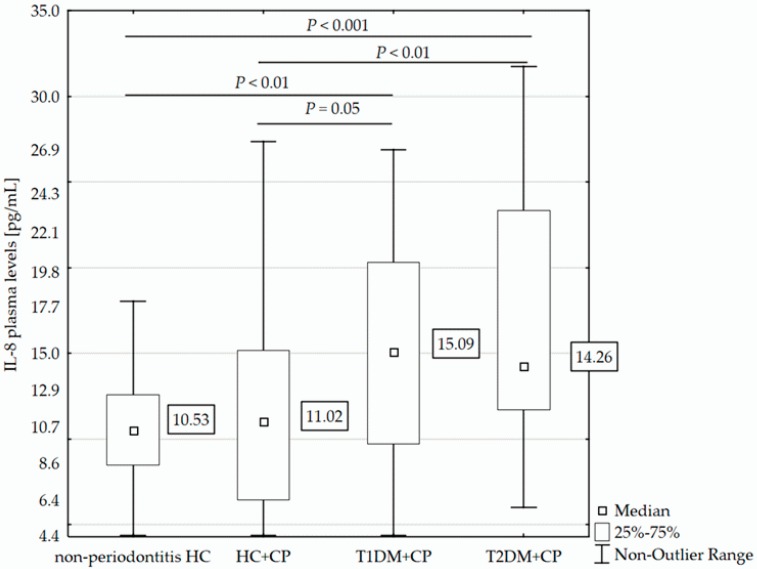
Comparison of IL-8 plasma levels in T1DM+CP (*N* = 36) and T2DM+CP patients (*N* = 44) and HC+CP (*N* = 41) and non-periodontitis HC (*N* = 32). Samples with IL-8 levels under the limit of detection (<4.40 pg/mL) were assigned a value of 4.39 pg/mL. The Mann-Whitney U-test (two-tail) was used for the calculation of significant differences.

Analysis of circulating IL-8 showed that plasma levels of this chemokine differed significantly between the T1DM+CP/T2DM+CP patients (median [interquartile range, IQR]: 15.09 pg/mL [9.73–20.32]/14.25 pg/mL [11.72–23.36], respectively) and non-periodontitis HC (10.53 pg/mL [8.48–12.58], *P* < 0.01 in both comparisons). Diabetic patients of both types with CP also had significantly higher levels of IL-8 than did HC+CP individuals (11.02 pg/mL [6.47–15.17], *P* ≤ 0.05). Interestingly, the groups of HC+CP and non-periodontitis HC and also the groups of patients with T1DM+CP and T2DM+CP always exhibited similar IL-8 plasma levels (see Figure 1). 

The IL-8 plasma levels in diabetic patients according to their glycemic control (as assessed by HbA1c levels) were comparable (*P* > 0.05, see Table 2). 

The comparison of IL-8 levels in T2DM patients of normal weight, those who were overweight, and obese showed no significant differences (*P* > 0.05, see Table 3).

In addition, concentrations of circulating IL-8 levels were not significantly associated with the level of glycemic control (blood glucose and HbA1c), smoking status, or clinical parameters such as GI, PD and AL (*P* > 0.05). However, increased IL-8 levels were detected in patients without DN (14.84 pg/mL [11.60–21.79]) in comparison to patients with DN (12.33 pg/mL [7.84–15.51]), *P* = 0.03).

### 2.3. IL-8 Plasma Levels and Gene Variability

The frequencies of both studied single nucleotide polymorphisms (SNPs), *IL-8* (−251A/T, rs4073) and *CXCR2* (+1208C/T, rs1126579) genotypes, in all subgroups were consistent with those expected from the Hardy-Weinberg equilibrium (HWE) (*P* > 0.05).

Allelic and genotype distributions were similar in all of the subgroups and the T2DM+CP group between well/satisfactorily controlled individuals vs. unsatisfactorily controlled patients (only data for genotype frequencies are shown). *IL-8* and *CXCR2* gene variability was also evaluated across the whole cohort in relation to IL-8 plasma levels. No association between the specific gene variants and circulating IL-8 levels was found (see Table 4). 

### 2.4. IL-8 Plasma Levels and Periodontal Bacteria

The microbial profiles were determined in a subgroup of 140 subjects. The participants were divided into two groups (negative and positive) for specific periodontal bacteria. Higher IL-8 plasma levels were found in the healthy controls and the well/satisfactorily controlled T2DM+CP patients positive for *T. forsythia* or *P. intermedia* than in individuals from this subgroup negative for these specific bacteria (*P* = 0.03 in both, *P_corr_* > 0.05). On the other hand, the presence of *P. gingivalis*, *T. forsythia*, or *T. denticola* was associated with lower IL-8 plasma levels in T1DM+CP patients (*P* < 0.01, *P_corr_* > 0.05).

*F. nucleatum* was detected in almost all of the individuals (98.6%). IL-8 levels were found to be higher in the absence of *F. nucleatum* than in the presence of this bacterium in HC+CP individuals (mean 46.40 pg/mL vs. 10.80 pg/mL), and in unsatisfactorily controlled T2DM+CP patients (mean 30.86 pg/mL vs. 13.77 pg/mL). However, the significance of these results is questionable due to the low number of patients who tested negative for *F. nucleatum* (only two individuals in total; see Table 5).

## 3. Discussion

CP and DM are common, multifactorial diseases worldwide [31]. Current evidence suggests that the relationship is bidirectional: DM increases the risk and severity of periodontitis, and periodontal disease can adversely affect the outcome of diabetes [32]. A potential link between DM and CP involves a broad axis of inflammation, a specific immune cell phenotype, serum lipid levels, and tissue homeostasis [33]. Changes in immune cell function in diabetic patients are reflected in the upregulation of proinflammatory cytokines and chemokines, such as the neutrophils chemoattractant IL-8 [34].

In this study, we evaluated the circulating levels of IL-8 as well as genetic and microbial profiles in systemically healthy individuals with or without CP and T1DM/T2DM patients with CP. The participants were selected from a large pool of patients and controls; in all groups, representation of both genders was balanced. The average ages of the non-periodontitis HC and T1DM+CP patients and also HC+CP and T2DM+CP patients were similar. The difference in the average age between patients with T1DM and T2DM was caused by divergent pathogenesis (age of onset) of the two types of the disease. Nevertheless, patients with T1DM were affected on average by a 15-year longer duration of this disease compared to those with type 2. Simultaneously, patients with T1DM+CP had more poorly controlled HbA1c levels than T2DM+CP patients. Periodontitis progression has recently been associated with elevated HbA1c levels in T2DM patients, and the treatment of periodontal infection may thus improve glycemic control of diabetic patients [35]. In this study, however, periodontal status, evaluated according to PD and AL, was similar between the CP patients and DM patients of both types with CP. Therefore, our results are in contradiction to other published articles which conclude that diabetic patients with difficulties in controlling serum glucose levels are more likely to suffer from periodontitis than well-controlled diabetic patients or individuals without diabetes [36]. This may be due to the relatively small sample size included in this study.

Based on the finding that high glucose-induced oxidative stress increases IL-8 production in human gingival epithelial cells, Kashiwagi et al. hypothesized a potential involvement of epithelial cells in periodontal disease during diabetes, caused by evoking an excessive host inflammatory response [22]. We found that IL-8 plasma levels were significantly higher in both diabetic groups than in HC+CP/non-periodontitis HC individuals. We assume that the differences in mean age between the groups did not affect the results. Even though IL-8 plasma levels were previously found higher in healthy older people than in healthy younger people, there was no statistical significance of this result [37]. Recent studies have shown that IL-8 is secreted by adipocytes, that circulating IL-8 levels in obese subjects without diabetes are significantly higher than in subjects with normal body weight, and that circulating levels of IL-8 are thus positively correlated with BMI [38]. In this study, no significant differences were found in IL-8 levels among T2DM normal weight, overweight, or obese subjects. However, this can be due to the low number of T2DM patients with normal body weight. While Engebretson et al. and Mohamed et al. reported significantly higher concentrations of IL-8 in GCF in patients with T2DM+CP as compared to HC+ CP [23,25], IL-8 GCF levels did not correlate with the diabetic status in the recent study by Longo et al. [24]. In contrast to our results, IL-8 circulating levels were found to be significantly lower in patients with T1DM than in first-degree control relatives [26]. Purohit et al. hypothesized that higher levels of this cytokine might be partly responsible for the protection against the development of T1DM [26]. On the other hand, Lappin et al. also found elevated plasma levels of IL-8 in T1DM+CP patients [21], and thus IL-8 may contribute to the cross-susceptibility between CP and DM. In addition, plasma levels of IL-8 were found to be similar in non-periodontitis HC and the HC+CP individuals in the Czech population. Nevertheless, in previous studies, higher plasma/serum levels have been associated with CP in different populations [21,39]. Frederiksson suggested that patients with CP had a subpopulation of peripheral neutrophils with a higher responsiveness to IL-8 priming than controls with healthy gingiva [40].

No association was found between circulating IL-8 level and glycemic control (blood glucose and HbA1c), smoking status, and clinical parameters such as PD and AL in the Czech population. In contrast, Lappin et al. reported that plasma levels of IL-8 correlated with levels of blood glucose and HbA1c, PD, and AL [21]. It should be noted that mean numbers of sites/teeth with PD/AL ≥ 5 mm in Czech patients were more than double of those in patients with CP and T1DM+CP in the study conducted by Lappin et al. [21]. The severity of periodontal tissue damage and inflammation may thus contribute to the differences in results. Surprisingly, higher IL-8 levels in patients without DN than in patients with DN were found in the studied cohort. This is not in accordance with the study by Perlman et al., who found an elevated transcript level of IL-8 at all DN disease stages as compared to controls [41]. Our results must be treated with caution because the number of patients with DN was very low (only 17 patients).

A further aim of this study was to correlate IL-8 circulating levels with variants in the *IL-8* and *CXCR2* genes. *IL-8* polymorphisms (−251A/T, rs4073) had previously been associated with changes in transcriptional activity [42]; namely, the A allele was linked with an increased IL-8 production in response to whole blood stimulation with lipopolysaccharides (LPS) [27]. In line with the results presented by Li et al. [39], no correlation of IL-8 plasma levels with the gene variants in *IL-8* was found in this study. We assume that no individual SNPs in the *IL-8* gene, but rather a combination of multiple variants, may affect the protein expression. Benakanakere et al. provide evidence that carriage of the *IL-8* ATC/TTC haplogenotype may increase the influx of neutrophils into inflammatory lesions and influence disease susceptibility [43]. Although synergistic interaction was observed between the T allele of the *CXCR2* (+1208C/T, rs1126579) SNP and high IL-8 serum levels [29], there were no differences among single variants and plasma levels of IL-8 in the present study. The current results of the genetic analysis were analogous with those from our previous research into *IL-8* gene variability in CP and aggressive periodontitis patients in a larger population [30]. Recent work by Nibali et al. claimed that host genetic variants could affect the colonization by specific microbes [44]. However, this could not be confirmed in this study due to the small size of the studied population.

Finally, the relationship between the microbial profile and chemokine levels was evaluated, but the presence of individual periodontopathic bacteria was not significantly associated with IL-8 production. Mesia et al. demonstrated that T2DM+CP individuals had higher unstimulated and stimulated levels of several cytokines, including IL-8, than systemically healthy individuals with periodontal disease [45]. In addition, *P. gingivalis* LPS-induced levels of IL-8 and others strongly correlated with disease severity (as measured by PD) in the T2DM group, but not in the group of controls with periodontitis [45]. While Lappin et al. found that *P. gingivalis* LPS and AGE together influenced the expression of IL-8 more than LPS alone in vitro [21], there were no differences in the presence of this bacterium between systemically healthy individuals and DM patients with similar periodontal status in the Czech population. Interestingly, *F. nucleatum* was detected in 98.6% of participants and higher IL-8 levels were recorded in patients without this bacterium in the oral cavity. This is inconsistent with previous findings, which showed that various *F. nucleatum* strains produce higher IL-8 levels in comparison with other oral bacteria [46,47]. The mechanisms behind these observations are still not well understood. Furthermore, our study only found two patients who were negative for *F. nucleatum*, one was from the group HC+CP and the other suffered from T2DM+CP.

The main limitation of our study is that subgroups of T1DM/T2DM without periodontal disease could not be included because only diabetic patients with a different severity of periodontitis were in the selected cohort. On the other hand, we can claim high homogeneity of the studied population because the participants were collected from a pool of patients of Czech Caucasians of European origin from South Moravia. The size of our cohort (*N* = 109) is comparable with the number of participants (*N* = 104) in the recent study by Lappin et al. [21]; moreover, we included an additional 44 T2DM+CP patients. Lappin et al. associated the elevated plasma levels of IL-8 with CP and/or T1DM and found that *P. gingivalis* LPS and AGE together caused a significantly greater expression of IL-8 from THP-1 monocytes and OKF6/TERT-2 cells than LPS alone. While no in vitro stimulation of cells by bacterial LPS or AGE was carried out in connection with IL-8 in our previous study [48], the advantage of the present study is that the Czech participants underwent detailed clinical, genetic, and microbiological examinations.

In conclusion, increased circulating levels of IL-8 were associated with DM of both types in the presence of periodontal disease, which suggests their important role in the pathogenesis of T1DM, T2DM, and CP. Nevertheless, the influence of single genetic variants of *IL-8*/*CXCR2* or the influence of the presence of individual periodontal bacteria on the concentrations of circulating IL-8 were not confirmed. Our findings may have some diagnostic implications and their molecular basis needs to be further addressed. Nevertheless, the design of the association study can be sensitive to type II statistical error and our results require confirmation by other studies on different populations.

## 4. Materials and Methods

The study was performed with the approval of the Committees for Ethics (15/2009) of the Medical Faculty, Masaryk University Brno and St. Anne’s Faculty Hospital. Written informed consent was obtained from all participants in line with the Declaration of Helsinki before inclusion into the study.

### 4.1. Study Population and Clinical Examinations

One hundred and fifty-three unrelated Caucasian adult participants from the South Moravian Region of the Czech Republic were included in this study. The periodontal status was evaluated in all individuals as follows: 73 systemically healthy individuals and 80 diabetic patients at the Clinic of Stomatology, Institution Shared with St. Anne’s Faculty Hospital, Faculty of Medicine, Masaryk University, Brno from 2010 to 2017. The diagnosis of periodontitis/non-periodontitis was based on detailed clinical examination, medical and dental history, tooth mobility and radiographic assessment. All patients with CP fulfilled the diagnostic criteria defined according to AL levels by the International Workshop for a Classification of Periodontal Diseases and Conditions for Chronic Periodontitis [49]: ≥30% of the teeth were affected (generalized CP) and PD was ≥4 mm. PD and AL were collected with a UNC-15 periodontal probe from four sites on every tooth present by an experienced periodontist.

For comparability with the study by Lappin et al. [21], the numbers of sites and teeth with PD ≥ 5 mm and AL ≥ 5 mm were recorded in both cases and controls. Loss of the alveolar bone was determined radiographically. Examination of lipid level profiles was as previously described [50]. The degree of gingival inflammation was assessed using the GI according to Löe and Silness [51], and the presence of inflammation of the gingiva was evaluated on four surfaces of all of those teeth present (distal, vestibular, mesial, oral). This index uses a 0–3 scale according to the following criteria: The complete absence of visual signs of inflammation was scored as 0; slight change in colour, slight oedema and no bleeding on probing as 1; and visual inflammation, redness, oedema, glazing, and bleeding on pressure as 2. Finally, severe inflammation, marked redness, oedema, ulceration, and the tendency to spontaneous bleeding was scored as 3. Using all of the individual scores, mean GI scores ± SD were calculated.

The diagnosis of T1DM (number of patients, *N* = 36) or T2DM (*N* = 44) was based on the presence of clinical symptoms (such as polyuria, polydipsia, and weight loss) and biochemical parameters (glycemia, glycated hemoglobin, ketoacidosis, and autoantibody status) in the outpatient unit of the Diabetology Clinics in Brno by experienced diabetologists. In accordance with American Diabetes Association guidelines [52], in patients with typical symptoms, the diagnosis was established upon finding glucose >11.0 mmol/L. In the absence of clinical manifestations, the diagnosis was made based on the finding of fasting blood glucose in venous plasma ≥7.0 mmol/L after 8 h of fasting, and the finding of blood glucose in venous plasma >11.0 mmol/L, or HbA1c ≥ 48 mmol/mol 2 h after the consumption of 75 g glucose (oral glucose tolerance test). Diabetes control was assessed as good (<45 mmol/mol), satisfactory (45–60 mmol/mol), or unsatisfactory (>60 mmol/mol) [53]. In addition, the presence of diabetic complications (such as DR, DPN, and DN), the duration of diabetes, and other clinical and biochemical parameters (BMI, smoking, lipid profile, etc.) were recorded.

The inclusion criteria for this study were the willingness to participate, compliance with the diagnostic criteria for CP and/or DM, and, for the control group, systemic and periodontal health. None of the participants were receiving treatment for periodontitis at the time of diagnosis but all were offered treatment whether they agreed, declined to participate, or were excluded from the study. All patients were firstly examined by a periodontist and they did not receive scaling and/or root planing minimally six months before measuring periodontal indices. The exclusion criteria for this study were a history of systemic diseases such as coronary artery diseases, malignancies, immunodeficiency disorders, current pregnancy or lactation, immunosuppression attributable to medication or concurrent illness, the use of antibiotics or anti-inflammatory drugs within six weeks of recruitment, <20 teeth (only in healthy controls), and the inability to consent.

### 4.2. Sample Collection and Plasma Levels Analysis

Levels of IL-8 in plasma were measured in all 153 individuals at the Department of Biochemistry, Faculty of Medicine, Masaryk University, Brno. The plasma samples were prepared from venous blood collected into a tube with EDTA (S-Monovette^®^ 9 mL K3E, Sarstedt, Germany), separated by centrifugation (465× *g*, 4 °C, 10 min), and stored at −70 °C within 30 min of collection.

IL-8 plasma levels were determined using enzyme-linked immunosorbent assay (ELISA) kits [IL-8 Human Magnetic Kit for Luminex™ Platform (Catalog No. LHC0081M, Novex™, Life Technologies, Grand Island, NY, USA) with Human/Monkey Extracellular Protein Buffer Magnetic Reagent Kit (Catalog No. LHB0001M, Novex™, Life Technologies, Grand Island, NY, USA)] and software (Luminex 200^TM^ analyzer with xPONENT 3.1 Software, Luminex Corporation, USA; Milliplex^TM^ Analyst v 3.4 Software, VigeneTech, Carlisle, MA USA) according to the manufacturer’s instructions. Those samples with IL-8 values under the limit of detection (<4.40 pg/mL) were arbitrarily assigned a value of 4.39 pg/mL for the statistical analysis.

### 4.3. Genetic Analysis

Genomic DNA was isolated from peripheral blood according to the standard protocol (phenol-chloroform method) by Sambrook et al. [54] and archived in the DNA bank at the Department of Pathophysiology, Faculty of Medicine, Masaryk University, Brno.

Two SNPs [*IL-8* (−251A/T, rs4073) and *CXCR2* (+1208C/T, rs1126579)] were genotyped using the fluorescent probes for allelic discrimination (TaqMan^®^ assays, Life Technologies, Grand Island, NY, USA; C_11748116_10 and C_8841198_10, respectively). A sequence detection system ABI PRISM 7000, Applied Biosystems, Waltham, MA, USA was used. Polymerase chain reaction conditions were 95 °C for 10 min and 40 cycles of 95 °C for 15 s and 60 °C for 1 min. Real-time and endpoint fluorescence data were analyzed by SDS version 1.2.3, Applied Biosystems, USA software. Ten percent of the samples were determined in duplicates with 100% accordance. Genotyping was performed by investigators unaware of the phenotype.

### 4.4. Periodontal Bacteria Analysis

Samples of subgingival microflora were collected from the deepest sulcus/pocket from each quadrant in oral cavity. After careful removal of supragingival plaque and drying, a sterile endodontic pin (ISO size 40) was introduced to the bottom of the periodontal sulcus for 10 s. After removal, the pins were inserted into a sterile transport tube and sent for evaluation.

Seven oral bacteria were investigated by the DNA microarray detection kit (Protean Ltd., Ceske Budejovice, Czech Republic), as published previously [30]. Briefly, individual bacteria were determined semi-quantitatively as follows: (−) undetected, which corresponds to bacteria count less than 10^3^; (+) slightly positive, corresponding to bacteria count of 10^3^–10^4^; (++) positive, corresponding to bacteria count of 10^4^–10^5^; and (+++) strongly positive, with bacteria count higher than 10^5^. Subgingival bacterial colonization (*Fusobacterium nucleatum, Aggregatibacter actinomycetemcomitans*, *P. gingivalis*, *T. forsythia*, *Treponema denticola*, *Prevotella intermedia*, *Parvimonas micra*) in subgingival sulci/pockets was analyzed in the subgroups of non-periodontitis HC (*N* = 19), HC+CP (*N* = 41), T1DM+CP (*N* = 36) and T2DM+CP patients (*N* = 44) before subgingival scaling. The diagnosis of the specific bacterial infection was assessed as positive when the number of bacterial cells exceeded 10^3^.

### 4.5. Statistical Analysis

In accordance with the “case–control” design of this study, “controls” (systemically healthy people without CP, so called non-periodontitis HC) were compared with “cases” (all patients with CP regardless of their diabetes status: HC+CP, T1DM+CP or T2DM+CP patients). Secondly, we used a “case–case” design to compare systemically healthy individuals and T1DM or T2DM patients with a similar periodontal status, as well as to make comparisons between patients with both types of diabetes.

Standard descriptive statistics were calculated: absolute and relative frequencies for categorical variables, mean with SD or median with quartiles for quantitative variables. One–way analysis of variance (ANOVA) and Kruskal–Wallis test (ANOVA) were carried out to compare continuous variables. The allele frequencies were calculated from the observed numbers of genotypes. The differences in the allele frequencies were tested using the Fisher’s exact test; HWE and genotype frequencies were calculated with the chi-square test. The association was described by odds ratios with 95% confidence intervals. Only the values of *P* < 0.05 were considered as statistically significant. Where appropriate, Bonferroni correction was used to adjust the level according to the number of independent comparisons to the overall value of 0.05. The adjusted *P* values are denoted as *P_corr_*. Statistical power calculation was based on the results from the previous study on plasma IL-8 levels by Lappin et al. [21]. To obtain an excess of 80% statistical power in an ANOVA with a detectable difference of 1.2 and mean of 1, a minimum of 17 samples was required in each of the four patient groups [21]. Similar to the previous study, we also increased the number to a minimum of 19 per group because the data did not conform to the normal distribution. Statistical analysis was performed using the statistical package Statistica v. 12, StatSoft Inc., Tulsa, OK, USA.

## Figures and Tables

**Table 1:** Demographic data for the studied T1DM+CP and T2DM+CP patients as well as HC+CP and non-periodontitis HC.

Characteristics (mean ± SD)	Non-Periodontitis HC *N* = 32	HC+CP *N* = 41	T1DM+CP *N* = 36	T2DM+CP *N* = 44
Age (years)	45.5 ± 9.6	59.5 ± 9.3 *	49.7 ± 10.4	66.8 ± 8.5 *
Duration of DM (years)	0.0 ± 0.0	0.0 ± 0.0	25.1 ± 11.8	10.2 ± 7.7
DM control (well/satisfactorily/unsatisfactorily controlled) %	−	−	0.0/8.3/91.7	7.7/28.2/64.1
Sex (males) %	31.3	26.8	44.4	40.9
Smoking (yes) %	5.3	19.4	25.0 *	7.1 ^‡,§^
DN (yes) %	0.0	0.0	27.6	16.7
DR (yes) %	0.0	0.0	63.3 ^‖^	0.0
DPN (yes) %	0.0	0.0	50.0 ^‖^	9.3
BMI	23.8 ± 4.2	26.8 ± 3.9	25.1 ± 3.1	29.9 ± 7.7 *^,§^
GI	0.3 ± 0.2	0.9 ± 0.3 ^†^	1 ± 0.3 ^†^	1.1 ± 0.3 ^†^
PD (mm)	0.6 ± 0.2	3.3 ± 0.9 *	3.6 ± 0.9 ^†^	3.8 ± 1.0 ^†^
AL (mm)	0.8 ± 0.2	4.1 ± 1.1 ^†^	4.4 ± 1.5 ^†^	4.7 ± 1.2 ^†^
N of sites with PD ≥ 5 mm	0	18 ± 17 *	20 ± 19 *	20 ± 16 *
N of teeth with PD ≥ 5 mm	0	10 ± 7 *	11 ± 7 *	11 ± 6 *
N of sites with AL ≥ 5 mm	0	32 ± 21 *	38 ± 28 *	38 ± 19 *
N of teeth with AL ≥ 5 mm	0	15 ± 7 *	15 ± 7 *	15 ± 6 *
HbA1c (mmol/mol)	-	-	69.6 ± 12.0	57.7 ± 14.5 ^§^
Blood glucose (mmol/L)	5.4 ± 0.5	5.7 ± 0.3	7.5 ± 2.3	7.7 ± 2.4
Total cholesterol (mmol/L)	-	-	4.7 ± 0.7	4.7 ± 1.0
Triglycerides (mmol/L)	-	-	1.0 ± 0.6	2.0 ± 1.2
LDL (mmol/L)	-	-	2.7 ± 0.6	2.7 ± 0.9
HDL (mmol/L)	-	-	1.6 ± 0.4	1.3 ± 0.3

− = unknown. Note: In T1DM+CP patients, the diabetic control was known only in 31 of them. * *P* < 0.05 in comparison to non-periodontitis HC. ^†^ *P* < 0.01 in comparison to non-periodontitis HC. ^‡^ *P* < 0.05 in comparison to HC+CP individuals. ^§^ *P* < 0.05 in comparison to T1DM+CP patients. ^‖^ *P* < 0.01 in comparison to T2DM+CP patients. DM: diabetes mellitus; DN: diabetic nephropathy; DR: diabetic retinopathy; DPN: diabetic neuropathy; BMI: body mass index; GI: gingival index; PD: pocket depth; AL: attachment loss; LDL: low density lipoprotein; HDL: high density lipoprotein.

**Table 2:** Comparison of IL-8 plasma levels in diabetic patients according to their glycemic control.

IL-8 Levels, Median [IQR] in pg/mL		
Well Controlled *N* = 4	Satisfactorily Controlled *N* = 14	Unsatisfactorily Controlled *N* = 57
12.68 [10.52–40.56]	14.45 [12.15–23.13]	14.04 [10.05–19.67]

Note: In T1DM+CP patients, the diabetic control was known only in 31 of them. IQR: interquartile range.

**Table 3:** Comparison of IL-8 plasma levels in T2DM patients according to their BMI.

IL-8 Levels, Median [IQR] in pg/mL		
Normal Weight BMI ≤ 25.0 kg/m^2^ *N* = 9	Overweight 25 kg/m^2^ < BMI ≤ 30 kg/m^2^ *N* = 14	Obese BMI > 30 kg/m^2^ *N* = 21
12.68 [9.16–16.54]	13.75 [12.00–23.13]	14.04 [12.50–16.24]

IQR: interquartile range.

**Table 4:** IL-8 plasma levels and polymorphisms in *IL-8* and *CXCR2* genes *.

SNPs Genotypes		IL-8 Levels, Median [IQR] in pg/mL											
		Non-Periodontitis HC *N* = 32 (%)		HC+CP *N* = 41 (%)		T1DM+CP *N* = 36 (%)		T2DM+CP *N* = 43 (%)		T2DM+CP Subgroups			
										Well/Satisfactorily Controlled *N* = 16 (%)		Unsatisfactorily Controlled *N* = 27 (%)	
*IL-8* (−251A/T, rs4073)	TT	10 (31.3)	9.20 [8.61–11.86]	10 (24.4)	10.36 [6.47–13.60]	11 (30.6)	13.16 [10.43–19.67]	16 (37.2)	13.75 [10.73–25.39]	5 (31.3)	12.87 [12.29–24.63]	11 (40.7)	14.33 [9.16–26.14]
	AT	15 (46.9)	11.00 [7.52–12.87]	21 (51.2)	7.68 [5.66–15.17]	16 (44.4)	17.38 [7.01–20.92]	17 (39.5)	14.18 [11.43–16.54]	6 (37.4)	15.10 [9.86–20.27]	11 (40.7)	13.49 [11.43–16.54]
	AA	7 (21.8)	10.86 [8.61–12.66]	10 (24.4)	12.19 [8.88–16.24]	9 (25.0)	13.33 [10.86–22.70]	10 (23.3)	15.80 [12.33–23.13]	5 (31.3)	16.69 [15.36–23.13]	5 (18.6)	14.04 [12.00–16.24]
*CXCR2* (+1208C/T, rs1126579)	CC	9 (28.1)	11.28 [8.61–12.87]	14 (34.1)	12.30 [7.92–15.21]	9 (25.0)	17.89 [10.43–20.87]	17 (39.5)	15.09 [12.29–23.91]	7 (43.8)	15.36 [12.29–24.63]	10 (37.0)	14.88 [9.16–23. 91]
	CT	17 (53.1)	9.09 [7.52–11.43]	18 (43.9)	11.57 [6.47–15.17]	20 (55.6)	14.00 [8.63–23.52]	21 (48.9)	13.16 [11.43–16.24]	7 (43.8)	14.18 [9.86–30.99]	14 (51.9)	13.16 [11.43–16.24]
	TT	6 (18.8)	11.43 [10.86–12.50]	9 (22.0)	6.99 [5.66–11.02]	7 (19.4)	12.00 [4.39–18.57]	5 (11.6)	16.69 [13.49–20.27]	2 (12.4)	18.48 [16.69–20.27]	3 (11.1)	13.49 [12.00–31.74]

* Genotypes are known only in 152 participants as one DNA sample was of poor quality.

**Table 5:** IL-8 plasma levels and periodontal bacteria in a subgroup of 140 individuals.

Bacteria (%)	IL-8 Levels, Median [IQR] in pg/mL											
	Non-Periodontitis HC *N* = 19		HC+CP *N* = 41		T1DM+CP *N* = 36		T2DM+CP *N* = 44		T2DM+CP Subgroups			
									Well/Satisfactorily Controlled *N* = 17		Unsatisfactorily Controlled *N* = 27	
*F. n.* neg	0.0	-	2.4	46.40 [46.40–46.40]	0.0	-	2.3	30.86 [30.86–30.86]	0.0	-	3.7	30.86 [30.86–30.86]
*F. n.* pos	100.0	10.21 [8.61–12.66]	97.6	10.80 [6.43–14.83]	100.0	15.09 [9.73–20.32]	97.7	14.18 [11.43–23.13]	100.0	15.36 [12.29–23.13]	96.3	13.77 [11.43–16.54]
*A. a.* neg	68.4	10.21 [8.61–12.50]	48.8	7.80 [5.99–13.32]	55.6	18.41 [10.38–24.41]	38.6	13.49 [12.33–16.54]	29.4	28.16 [12.87–30.99]	44.4	13.33 [10.58–14.50]
*A. a.* pos	31.6	10.14 [9.09–13.49]	51.2	13.45 [8.75–15.17]	44.4	13.25 [8.63–17.72]	61.4	15.09 [11.18–23.58]	70.6	14.77 [10.52–18.48]	55.6	15.09 [12.00–26.14]
*P. g.* neg	68.4	9.73 [7.24–12.66]	14.6	13.18 [7.99–27.35]	25.0	24.33 [16.86–24.78]	22.7	14.26 [12.33–26.14]	17.6	12.33 [9.09–15.36]	25.9	16.24 [12.50–30.86]
*P. g.* pos	31.6	10.54 [9.41–12.50]	85.4	10.57 [6.02–14.48]	75.0	12.00 [7.24–18.92] *	77.3	14.26 [11.43–23.13]	82.4	16.36 [12.29–24.63]	74.1	13.77 [10.58–15.82]
*T. f.* neg	36.8	8.61 [6.72–9.73]	2.4	46.40 [46.40–46.40]	8.3	24.33 [19.67–40.67]	0.0	-	0.0	-	0.0	-
*T. f.* pos	63.2	11.67 [9.81–13.66] *	97.6	10.80 [6.43–14.83]	91.7	13.33 [9.41–19.43] *	100.0	14.26 [11.72–23.36]	100.0	15.36 [12.29–23.13]	100.0	14.04 [11.43–23.58]
*T. d.* neg	47.4	8.77 [7.24–12.33]	14.6	14.83 [13.45–19.37]	25.0	20.87 [19.43–24.78]	22.7	13.33 [12.50–15.36]	17.6	12.87 [12.33–15.36]	25.9	13.49 [12.50–16.54]
*T. d.* pos	52.6	10.93 [9.41–13.83]	85.4	10.15 [6.39–14.04]	75.0	12.00 [7.24–18.57] *	77.3	14.50 [11.18–23.91]	82.4	16.36 [11.18–24.63]	74.1	14.19 [10.58–23.75]
*P. m.* neg	31.6	9.89 [7.24–12.66]	2.4	46.40 [46.40–46.40]	0.0	-	0.0	-	0.0	-	0.0	-
*P. m.* pos	68.4	10.21 [9.09–12.50]	97.6	10.80 [6.43–14.83]	100.0	15.09 [9.73–20.32]	100.0	14.26 [11.72–23.36]	100.0	15.36 [12.29–23.13]	100.0	14.04 [11.43–23.58]
*P. i.* neg	47.4	8.77 [6.72–12.66]	41.5	7.99 [6.02–14.48]	41.7	17.89 [12.15–24.33]	45.5	14.08 [10.14–16.39]	35.3	11.76 [9.09–15.36]	51.9	15.05 [11.43–23.58]
*P. i.* pos	52.6	10.54 [9.41–12.50]	58.5	11.57 [7.60–15.19]	58.3	12.00 [7.84–18.92]	54.5	14.26 [12.40–24.27]	64.7	20.27 [12.87–28.16] *	48.1	13.49 [12.00–14.67]

Neg: negative; pos: positive. * *P* < 0.05 in comparison of IL-8 plasma levels between patients negative and positive for the specific bacteria (*P_corr_* > 0.05).

## Abbreviations

*A. a.*	*Aggregatibacter actinomycetemcomitans*
AL	Attachment loss
ANOVA	One-way analysis of variance
BMI	Body mass index
CXCR2	C-X-C motif chemokine receptor 2 (receptor for IL-8)
CP	Chronic periodontitis
DM	Diabetes mellitus
DN	Diabetic nephropathy
DPN	Diabetic neuropathy
DR	Diabetic retinopathy
ELISA	Enzyme-linked immunosorbent assay
*F. n.*	*Fusobacterium nucleatum*
GCF	Gingival crevicular fluid
GI	Gingival index
HbA1c	Glycated hemoglobin
HC	Systemically healthy controls without periodontitis
HC+CP	Systemically healthy individuals with chronic periodontitis
HDL	High density lipoprotein
HWE	Hardy-Weinberg equilibrium
IL-8	Interleukin-8
IQR	Interquartile range
LDL	Low density lipoprotein
LPS	lipopolysaccharide
N	Number
Neg	Negative
PD	Pocket depth
*P. g.*	*Porphyromonas gingivalis*
*P. i.*	*Prevotella intermedia*
*P. m.*	*Parvimonas micra*
Pos	Positive
qPCR	Quantitative polymerase chain reaction
SD	Standard deviation
T1DM	Type 1 diabetes mellitus
T2DM	Type 2 diabetes mellitus
*T. d.*	*Treponema denticola*
*T. f.*	*Tanarella forsythia*
*P. i.*	*Prevotella intermedia*
